# Deep Brain Stimulation to Alleviate Freezing of Gait and Cognitive Dysfunction in Parkinson's Disease: Update on Current Research and Future Perspectives

**DOI:** 10.3389/fnins.2018.00029

**Published:** 2018-02-16

**Authors:** Chuyi Huang, Heling Chu, Yan Zhang, Xiaoping Wang

**Affiliations:** ^1^Department of Neurology, Shanghai TongRen Hospital, School of Medicine Shanghai, Jiao Tong University, Shanghai, China; ^2^Department of Neurology, Huashan Hospital, Fudan University, Shanghai, China

**Keywords:** freezing of gait, Parkinson's disease, deep brain stimulation, cognitive function, subthalamic nucleus, pedunculopontine nucleus

## Abstract

Freezing of gait (FOG) is a gait disorder featured by recurrent episodes of temporary gait halting and mainly found in advanced Parkinson's disease (PD). FOG has a severe impact on the quality of life of patients with PD. The pathogenesis of FOG is unclear and considered to be related to several brain areas and neural circuits. Its close connection with cognitive disorder has been proposed and some researchers explain the pathogenesis using the cognitive model theory. FOG occurs concurrently with cognitive disorder in some PD patients, who are poorly responsive to medication therapy. Deep brain stimulation (DBS) proves effective for FOG in PD patients. Cognitive impairment plays a role in the formation of FOG. Therefore, if DBS works by improving the cognitive function, both two challenging conditions can be ameliorated by DBS. We reviewed the clinical studies related to DBS for FOG in PD patients over the past decade. In spite of the varying stimulation parameters used in different studies, DBS of either subthalamic nucleus (STN) or pedunculopontine nucleus (PPN) alone or in combination can improve the symptoms of FOG. Moreover, the treatment efficacy can last for 1–2 years and DBS is generally safe. Although few studies have been conducted concerning the use of DBS for cognitive disorder in FOG patients, the existing studies seem to indicate that PPN is a potential therapeutic target to both FOG and cognitive disorder. However, most of the studies have a small sample size and involve sporadic cases, so it remains uncertain which nucleus is the optimal target of stimulation. Prospective clinical trials with a larger sample size are needed to systematically assess the efficacy of DBS for FOG and cognitive disorder.

## Introduction

Freezing of gait (FOG) is a gait disorder featured by recurrent transient gait retardation and interruption. FOG commonly occurs in Parkinson's disease (PD), primary progressive freezing gait, parkinsonism-plus syndromes and vascular parkinsonism. FOG is a major disabling feature of PD and usually occurs in late-stage PD with a severe impact on life quality of patients. FOG refers to the feeling of having the feet stuck to the floor and struggling to take a step. Most FOG episodes are related to the OFF state in PD, but severe cases begin to suffer from ON state freezing of gait (On-FOG) (Nutt et al., 2011). FOG increases the risk of falls for PD patients and has a large impact on the motor function and daily life of the patients. The pathogenesis of FOG is not fully known (Vandenbossche et al., 2013). Study has demonstrated that FOG is associated with cognitive disorder in PD patients. FOG and cognitive disorder are linked through mutual cause-effect relationship, and both conditions will deteriorate as the disease progresses (Vandenbossche et al., 2013). Medication therapy is the first choice for PD combined with either FOG or cognitive disorder, but the efficacy is limited (Giladi, 2008). Deep brain stimulation (DBS) has been regarded as a well-established, safe and effective surgical option for advanced stage PD which significantly improves the self-care ability of PD patients (Deuschl and Agid, 2013). Previous studies have confirmed that DBS can improve not only cognitive function, but also FOG; however, the controversy still goes on as to the efficacy of DBS (Kuhn et al., 2015; Schlenstedt et al., 2017). The disagreement between the research findings may arise from the differences in position and frequency of stimulation as well as the observation time. If DBS works by influencing the cognitive function in FOG, that means DBS can be used to treat the refractory symptoms in advanced PD combined with either FOG or cognitive disorder. Although this hypothesis may work in theory, insufficient scientific evidence is available nowadays. Therefore, this study mainly reviews the clinical trials on the efficacy of DBS in FOG of PD patients over the past decade. Also, the concurrent effect of DBS on cognitive functions was discussed based on the limited literature.

## Basic characteristics of FOG

### Definition

FOG is defined as “an episodic inability (lasting seconds) to generate effective stepping in the absence of any known cause other than parkinsonism or high-level gait disorders. It is most commonly experienced during turning and step initiation but also when faced with spatial constraint, stress, and distraction. Focused attention and external stimuli (cues) can overcome the episode” (Giladi and Nieuwboer, 2008).

### Incidence

FOG is most common in PD, especially late-stage PD. However, some PD patients already suffer from FOG episodes at an early stage (Lieberman et al., 2015). A retrospective cross-sectional study indicated that about 7% of the PD patients presented with FOG symptoms in the first 2 years, 28% of the PD patients in 5 years, 39% of the PD patients in 10 years, and 58% of the PD patients after 10 years (Giladi et al., 2001). A community-based prevalent cohort of 232 PD patients was followed-up prospectively over 12 years. The point prevalence of FOG at baseline was 27 and 63% of patients had developed FOG by the study end (Forsaa et al., 2015). Moreover, Perez-Lloret and colleagues demonstrated 38.2% of 652 PD patients reported FOG during the on state (Perez-Lloret et al., 2014). Apparently, a large proportion of the PD patients suffer from FOG.

### Clinical features

FOG is considered as the fifth major feature of PD after bradykinesia, tremor, dystonia and gait disorder. Typical clinical features of FOG include the followings: (1) FOG occurs predominantly in the OFF state. The risk of FOG is higher if the PD patients present with abnormal gait but no tremor at an early stage (Macht et al., 2007); (2) FOG symptoms occur suddenly, with a sense of having the feet stuck to the floor in spite of the inclination of the trunk to move forwards. However, FOG is sometimes alleviated by focusing or external stimulation (Giladi and Nieuwboer, 2008). The patients can resume normal or near-normal walking once they overcome the freezing; (3) There is a sense of having the front foot stuck to the floor, while the heel of the back foot suspended in the air, with the knees shaking alternatively at a frequency of 3–8 Hz; (4) Gait hesitation occurs concurrently (Nutt et al., 2011). Hesitation is most common during step initiation (start hesitation), walking through narrow passageway (hesitation in tight quarters) or turning (turning hesitation). The patients are unable to establish normal gaits. FOG usually happens unexpectedly, which leads to falls or injuries (Fasano and Bloem, 2013).

### Evaluation of FOG

The influence of FOG on the daily life of patients is usually accessed via rating scales. Unified Parkinson's disease rating scale (UPDRS) is most commonly used for PD patients. The score of the item related to FOG (item 14) is widely used to determine the severity of FOG. Gliadi et al. established the freezing of gait questionnaire (FOG-Q) in 2000, which is highly reliable in clinically assessing FOG (Giladi et al., 2000). New FOG-Q (NFOG-Q) includes relevant videos on the basis of FOG-Q, which are first played among the subjects before the assessment. This improved version of questionnaire enables the assessment in both ON and OFF state and determines the severity of FOG more accurately (Nieuwboer et al., 2009). However, the above scales are subjective measurements. Several objective methods have emerged in recent years, which use special equipment to detect relevant indicators of FOG. Moore and colleagues used an ankle-mounted sensor array for the vertical linear acceleration of the left shank which transmitted data wirelessly to a pocket PC. A freeze index was defined and the values above this limit were designated as FOG (Moore et al., 2008). In addition, gastrocnemius surface electromyograph could also be used to evaluate FOG (Wang et al., 2014).

## Pathogenesis of FOG

### FOG related brain regions and neural circuit

The pathogenesis of FOG remains unclear and injuries of the nervous system on different levels (spinal cord, cerebellum, brainstem, basal ganglia, thalamus, and cerebral cortex) can lead to FOG in PD patients (Grabli et al., 2012). Magnetic resonance imaging (MRI) and nuclear medical imaging have revealed significant changes in the structures between the motor cortex network and subcortical region as well as disruption of functional connections in FOG patients. Episodic gait disturbance may result from the acute neural network overload related to neural decomposition under motor conflicts or cognitive or emotional stimuli, including cognition, motor or even anxiety processing (Fasano et al., 2015a). It was demonstrated by significant gray atrophy in left temporal, right frontal and right cerebellum of PD patients with FOG accompanied by the impairment of these regions (Jha et al., 2015). Vercruysse et al. showed that FOG in PD patients was associated with diffuse white matter injury, which affected bilateral cerebellum, superior longitudinal fasciculus, right internal capsule, anterior corona radiata and left thalamic radiation (Vercruysse et al., 2015). The above studies have demonstrated that FOG in PD patients is combined with diffuse white and gray matter injuries that affect the motor, sensory and cognitive regions. The conditions make the patients prone to acute neural network overload under the above stimuli, which leads to episodic FOG. In addition, injuries of the connections between the left and right hemispheres can also cause FOG due to loss of control of the legs and incoordination (Lenka et al., 2016).

The pedunculopontine nucleus (PPN) is an important part of the midbrain motor area and projects to the basal ganglia, thalamus, cortex, brainstem, cerebellum and spinal cord. PPN has intimate fibrous connections with the basal ganglia and plays a role in regulating the gait and postures. FOG is considered relevant to the abnormalities in the functional connectivity of PPN and to the microstructural anomalies of the subcortical region (Nutt et al., 2011). One study suggests that FOG in PD is associated with abnormal PPN functional connectivity network, mainly affecting the corticopontine-cerebellar pathways and visual temporal areas (Wang et al., 2016). Moreover, Youn's work using diffusion tensor imaging (DTI) shows microstructural changes of PPN and connected subcortical structures such as basal ganglia, thalamus and cerebellum, which are closely related with FOG in PD patients. It is apparent that PPN plays a key role in the pathogenesis of FOG and serves as an important therapeutic target for DBS.

Both the ultra-direct and indirect pathways of the basal ganglia activate globus pallidus internalis (GPi) and substantia nigra pars reticulata (SNr) via the subthalamic nucleus (STN), thus inducing extensive inhibition of the excitability of the cerebral cortex. STN is considered as an important node in immobilization. Fling et al. found through resting-state functional MRI and DTI that the functional connectivity between STN and supplementary motor area (SMA) in PD patients with FOG decreased significantly as compared with the PD patients without FOG. It was inferred that the loss of functional connectivity of the STN-SMA circuit caused the loss of the ability in inhibiting competitive activity and initiating the right motion (gait), thus leading to FOG (Fling et al., 2014). Some scholars believe that the injury of different brain areas or neurological deficit can lead to the decline or loss of the information processing ability of basal ganglia, temporary excess activation of STN and increased inhibitory output of the basal ganglia. As a result, SMA and locomotor area of the midbrain are inhibited excessively. The above changes are the common mechanisms of the occurrence of FOG under different pathological conditions (Lewis and Shine, 2016).

Besides, cerebral cortical dysfunction is also related to FOG. PD usually involves the basal ganglia, which leads to decreased spontaneous motor activity. As a result, the areas of the brain involved in higher functions are recruited for gait function as a compensatory mechanism. That is why PD patients with FOG perform easy gaits with more exertions (Wu et al., 2004). Study has suggested that episodes of FOG are more likely to occur when PD patients do math while walking. Knobl et al. carried out an in-depth research concerning this topic and found that increased cognitive load facilitated FOG (Knobl et al., 2012). In frontal assessment battery, phonemic verbal fluency, Stroop test, and ten-point clock test, the scores of FOG patients were significantly lower than those of non-FOG patients (Amboni et al., 2008). The above results indicated that the impairment of the brain's higher functions is involved in FOG.

### Cognitive models of FOG

**Figure 1:**
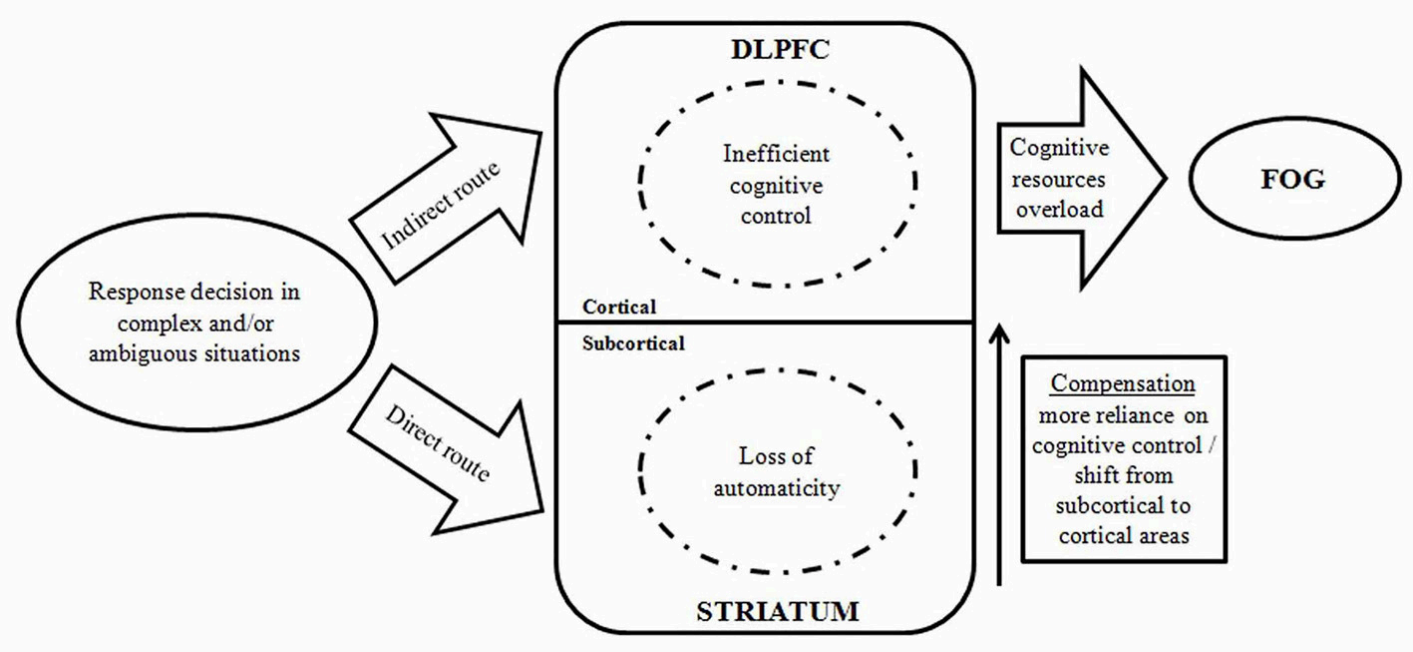
A model displaying the interaction between automatic and controlled cognitive dysfunctions in the occurrence of FOG episodes. (DLPFC, dorsolateral prefrontal cortex; FOG, freezing of gait) (Vandenbossche et al., 2013).

An animal model of dual cholinergic–dopaminergic losses to simulate falls associated with FOG in PD patients was established. This model impaired cognitive control of complex movements via disturbing cortico-striatal interactions. Falls and FOG were reduced with cognitive improvement by the treatment of an acetylcholinesterase inhibitor and a 5-HT_6_ receptor antagonist (Kucinski et al., 2017). Vandenbossche et al. proposed the cognitive model, which conceptualizes FOG into a specific impairment of conflict resolution and deterioration of executive functions (Figure 1; Vandenbossche et al., 2011, 2013). In a neuropsychological consistency test, both FOG and non-FOG patients displayed an impairment of conflict resolution mechanism. But compared with the normal control group, significant difference only existed in FOG patients (Vandenbossche et al., 2011). Another study demonstrates a strong incorrect response activation but a reduction in effect inhibition of conflicting responses in FOG patients (Vandenbossche et al., 2012). This situation becomes more conspicuous when the controlling input that compensates for this defect decreases. Thus, executive dysfunction promotes the occurrence of FOG. The striatal circuit, STN and right inferior frontal gyrus are considered to be relevant to the conflict resolving signaling pathway. That is, when conflict occurs, GPi threshold is increased to temporarily prevent early response and to prolong selected response until the conflict is resolved (Frank et al., 2007). Freezing induced by defect in the conflict resolving mechanism is related to the reduction in blood oxygen level-dependent response in the subcortical areas. This point of view confirms the cognitive model (Shine et al., 2013). Brain imaging studies have shown that the structural impairment of the frontal and parietal cortex and reduction in functional connectivity may be related to executive dysfunction in FOG patients. More studies are needed to confirm this.

## Therapeutic effects of DBS on FOG and the accompanied cognitive disorder

**Figure 2:**
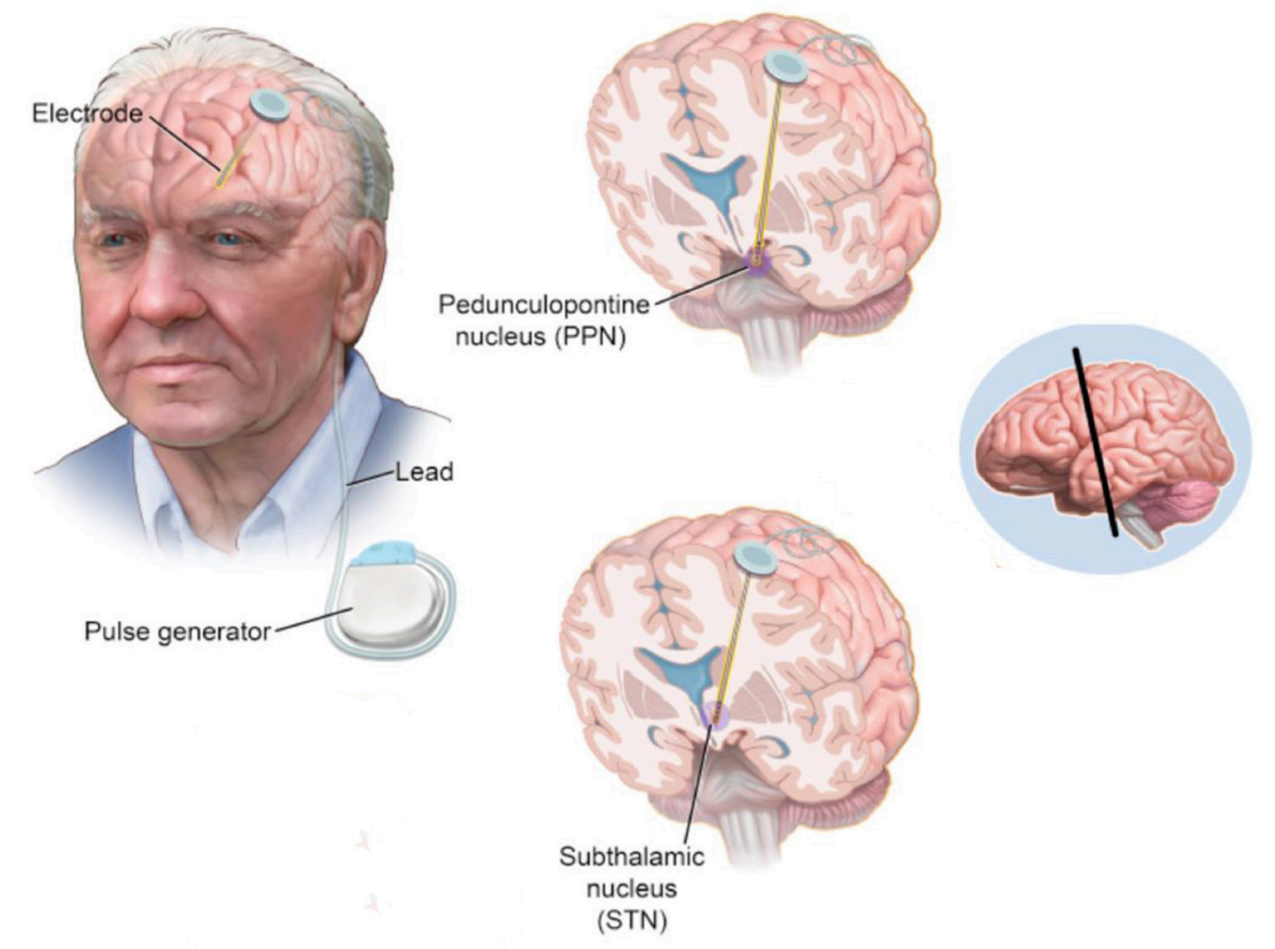
A schematic visualization of DBS on STN and PPN in a PD patient (Hickey and Stacy, 2016).

DBS produces mild, continuous electrical pulses to the neural nuclei of the brain. Compared with the ablative procedure, DBS has various advantages including minimally invasive, controllable, repeatable switching on and off and reversible side effects. DBS has been applied to treat PD since the first attempt of thalamus stimulation with high frequency by Dr. Alim-Louis Benabid in 1987. The surgical procedure of DBS includes the method of locating the surgical targets and implanting the leads and microelectrode(s). In summary, it starts with stereotactic localization of the target nucleus. Pre-operative stereotactic computed tomography (CT) scan fused with a cerebral MRI and microelectrode recording (intraoperative electrophysiological mapping) are performed to confirm accuracy of lead positioning. After the lead and microelectrode are secured, its position is then checked with postoperative MRI/CT scan. Microstimulation with the DBS lead is then performed with clinical observation. After DBS, the stimulation parameters and dopaminergic therapies are adapted postoperatively by a neurologist periodically. A schematic visualization of DBS on STN and PPN in a PD patient is shown in Figure 2.

### The effect of DBS on cognitive disorder of PD patients

The current study about the treatment of cognitive disorder by DBS shows that fornix and nucleus basalis of Meynert (NBM) are two main DBS targets which can improve the learning and memory capacities (Mirzadeh et al., 2016). For example, a pilot study of six patients revealed DBS to NBM ameliorated Alzheimer's disease associated symptoms accompanying cerebral glucose consumption improvement (Kuhn et al., 2015). Although STN and PPN stimulated by DBS are not the nuclei involved in cognitive improvement, an analysis on the projection neuron circuit indicates the potential efficacy of STN- and PPN-DBS in improving the cognitive disorder. It was demonstrated in hemiparkinsonian rats that 6-hydroxydopamine lesioning increased the levels of glutamate and gamma-amino butyric acid (GABA) in striatum and SNr, which could be normalized by chronic STN-DBS (Chassain et al., 2016). As glutamate and GABA are closely related to the memory function in PD patients (Buchanan et al., 2014), it suggests STN-DBS may influence the basal ganglia networks which are associated with cognitive functions. Meanwhile, since pedunculopontine tegmental nucleus (PPTg) acts as an interface between the basal ganglia and cerebellum, it has potentials to influence motor control as well as cognitive functions (Mori et al., 2016). Nevertheless, although it is likely that STN- and PPN-DBS are able to improve cognitive function based on the above theories, the studies concerning the long-term effects of STN- and PPN-DBS on cognitive impairment are very limited. It was even reported subthalamic stimulation may have cognitive side effects as a decrease in phonemic and semantic verbal fluency (Castrioto et al., 2014). Since cognitive function can be exacerbated along with age and disease progression, such side effects are still difficult to be evaluated without the inclusion of good control groups. More studies indicate that both GPi-DBS and STN-DBS produce subtle cognitive declines but appear to be relatively well tolerated and GPi-DBS seems to cause fewer neurocognitive declines than STN-DBS (Combs et al., 2015). Therefore, considering the close relation between FOG and cognition, DBS has potentials to improve both of the severe symptoms, which will be detailedly discussed in the following text.

### FOG treated by DBS

As analyzed above, FOG is closely related to the abnormalities of higher cortical functions. FOG may be understood as a gait-related symptom of cognitive dysfunction. Pallidotomy had been attempted as a therapeutic method at early stage. However, an increase in FOG and cognitive decline were observed after a 10-year follow-up (Hariz and Bergenheim, 2001). Currently, drug therapies, including levodopa, monoamine oxidase inhibitors, etc., are the main choice for FOG in PD patients (Zhang et al., 2016). A study evaluated the gait phenomenology of 20 PD patients with FOG before and 60 min after a standardized levodopa dose. Levodopa reduced the freezing sum score from a median of 15 to 3.5 (*p* < 0.0001). Moreover, those patients with lower pre-dose item-scores also showed lower post-dose outcome scores (Fietezk et al., 2013). Besides, scientists also find that some drugs, such as amantadine, L-threo-3, 4-dihydroxyphenylserine, and botulinum toxin have exhibited varying degrees of beneficial effects (Zhang et al., 2016). However, for many PD patients with concurrent FOG and cognitive disorder, the efficacy is poor using either a single drug or combined medication therapy. An increasing attention has been drawn to DBS treatment for FOG, which mainly involves the stimulation of STN and PPN. However, STN- and PPN-stimulation have not been used in cognitive improvement. It is generally accepted that STN and PPN are related to cognition in terms of neural circuit. Given the mutual interaction between FOG and cognitive function, we infer that the improvement of FOG may be related to cognitive improvement. Few studies have been conducted concerning the DBS treatment of FOG combined with cognitive disorder. Here we discussed the effect of DBS on FOG by the stimulation of either STN or PPN alone or in combination. The research findings will shed new light on the treatment of FOG combined with cognitive disorder. The representative literatures are listed in Table 1.

**Table 1:** Summary of published studies related to the effects of DBS on FOG and cognitive functions in PD patients.

**Studies**	**No. of patients**	**Mean age (y)**	**Duration of PD**	**Study design**	**Stimulation site**	**Stimulation frequency**	**Follow-up duration**	**Evaluation of FOG**	**Evaluation of cognition**	**Outcomes**
**STN STIMULATION**										
Nilsson et al., 2009	10	66	18 (10–22)	Randomized, double blind	Bilateral STN	100–185 Hz	At least 12 months	Clinical performance tests, fear of falling ratings, posturography		STN stimulation alone significantly increased the scores of the Berg balance and the total score of the Falls-Efficacy Scale.
Fasano et al., 2010	20	56.9	13.7 ± 4.8	Retrospective	Bilateral STN	130 Hz	8 years	UPDRS-III	MMSE, RPM '47, Corsi's block -tapping test forward and backward, digit span forward and backward, letter verbal fluency, RAVLT, MWSCT	The UPDRS score (item 29) was decreased by STN-DBS from 2.2 ± 1.0 (baseline) to 1.3 ± 1.3 (8 year follow-up) with a slight worsening of cognition.
Xie et al., 2012	2	64	11	Non-randomized, non-blind	Bilateral STN	60 Hz	10 months	UPDRS-III		Switching the frequency from 130 to 60 Hz immediately alleviated the FOG in both ‘off’ and ‘on’ statuses and the effect lasted at least 10 month.
Niu et al., 2012	10	61.7	12.0 ±2.3	Non-randomized, non-blind, prospective	Bilateral STN	185 Hz	6 and 12 months	UPDRS-III, FOG-Q	Mattis Dementia Rating Scale	DBS was associated with significant improvement in FOG score and neuropsychological function at both 6 and 12 months.
Rocchi et al., 2012	29	61.3	12.4	Randomized, double blind	Bilateral STN or GPi	77%:185 Hz; 23%: 130–150 Hz	6 months	UPDRS-III, anticipatory postural adjustments		Six months of DBS in the STN or GPi impaired anticipatory postural preparation for step initiation.
Sidiropoulos et al., 2013	45	59.5	17.8 ± 5.7	Non-randomized, non-blind	Bilateral STN	≤80 Hz	4 years	UPDRS-III		No significant improvement was found in total motor UPDRS scores, and axial and gait subscores.
Ramdhani et al., 2015	5	66	14	Retrospective review	Bilateral STN	60 Hz	2–6 months	UPDRS-III		Low frequency STN stimulation early in the DBS programming course revealed clinical efficacy in more advanced PD patients with levodopa responsive gait disturbance and FOG.
Vercruysse et al., 2014	41	58.2	12.1	Non-randomized, non-blind, prospective controlled	Bilateral STN	185 Hz	6 and 12 months	NFOG-Q, UPDRS-III		STN-DBS reduced FOG occurrence and severity at 6 months postsurgery with largely sustained effects at 12 months follow-up.
Phibbs et al., 2014	20	62	12.5 (5–22)	Randomized, double blind	Bilateral STN	60 or 130 Hz	No long term follow-up	UPDRS-III, SWS test, GaitRite gait evaluation		Two of the 20 patients reported a significant subjective improvement in their gait with no statistical difference in their outcomes.
Xie et al., 2015	7	64	12.9 ± 4.9	Randomized, double blind	Bilateral STN	60 Hz	6 weeks	UPDRS-III, FOG-Q, SWS test		Low-frequency stimulation significantly reduced aspiration frequency and perceived swallowing difficulty. It also significantly reduced FOG, and axial and parkinsonian symptoms.
Vallabhajosula et al., 2015	19	61.8	13.6 ± 4.2	Randomized, blind and non-blind portions	Bilateral STN	60 or >100 Hz	No long term follow-up	UPDRS-III, static and dynamic postural control, gait evaluations		Total UPDRS-III score, step length and velocity during gait initiation, and gait speed significantly improved during 60 and >100 Hz conditions. No significant differences between 60 and >100 Hz conditions.
Lizarraga et al., 2016	22	65	NA	Randomized, double blind	Right: 125.3 ± 27.50 Hz; Left: 123.4 ± 23.20 Hz		No long term follow-up	UPDRS-II, III		Bilateral STN-DBS yields greater improvement in motor and gait scores in PD patients. Yet, unilateral stimulation has similar effects on gait kinematics. Particularly, right-sided stimulation might produce slightly greater improvements.
Chenji et al., 2017	17	61.7	NA	Randomized, double blind	bilateral, unilateral left, unilateral right STN	NA	No long term follow-up	UPDRS-III, GaitRite		Bilateral STN-DBS was superior to unilateral for some gait parameters (step length and double-limb support time), and MDS-UPDRS motor scores.
Kim et al., 2017	112	NA	12.2	Non-randomized, non-blind	Bilateral STN	NA	12 months	UPDRS-III, FOG-Q		Preoperative depression negatively affects the outcome of FOG, following STN-DBS in the off-medication state
**PPN STIMULATION**										
Ferraye et al., 2010	6	63.3	20.7 ± 7.1	double-blind, cross-over	Bilateral PPN	15–25 Hz	12 months	UPDRS-II, III, FOG-Q		The duration of freezing episodes as well as falls related to freezing was improved by PPN-DBS. However, the overall results had no significantly change during the double-blind evaluation.
Thevathasan et al., 2010	11	64.5	11.6 (4–17)	Non-randomized, non-blind	Bilateral PPN	20–35 Hz	12.7 (2–38) months	UPDRS-III, FOG-Q		Acute PPN stimulation improved gait and balance but not akinesia scores. Chronic PPN stimulation significantly improved falls frequency.
Moro et al., 2010b	6	65.2	15.5 ± 6.2	Randomized, double blind	Unilateral PPN	50–70 Hz	3 and 12 months	UPDRS-II, III		Patients reported a significant reduction in freezing and falls in the on and off medication states both at 3 and 12 months after PPN-DBS.
Thevathasan et al., 2012	7	64.1	17.7	Non-randomized, non-blind	Unilateral or bilateral PPN	20–40 Hz	2–13 months	UPDRS-II, III, FOG-Q		Improvement of FOG was associated with attenuation of alpha activity detected by electroencephalography.
Welter et al., 2015	4	NA	NA	Randomized, double blind, cross-over	Bilateral PPN	5–130 Hz	4 and 6 months	RSGE, UPDRS-II, III	MDRS, Phonological Fluency test, Trail Making test, Continuous Performance test, Stroop Task, FCSRT, ROCF copying test	Combination of PPN-DBS and levodopa treatment produced a significant decrease of the freezing episodes and the frequency of falls. No significant changes were observed in cognitive functions.
Mestre et al., 2016	9	63	15 (11–20)	Randomized, double blind	Unilateral PPN	60–70 Hz	2 and 4 years	UPDRS-II, III		At 2 years, patient-reported freezing was significantly better by PPN-DBS, while at 4 years, there was no significant change at 4 years.
**COMBINED STIMULATION**										
Stefani et al., 2017	6	64.5	12.1 ± 3.0	Randomized, double blind	Bilateral STN and Bilateral PPN	STN: 130–185 Hz; PPN: 25 Hz	3–6 months	UPDRS-II, III, S&E		PPN-DBS associated with standard STN-DBS improved gait and postural items of UPDRS-III.
Peppe et al., 2010	5	57.8	16.0 ± 10.0	Non-randomized, non-blind	Bilateral STN and Bilateral PPTg	STN: 185 Hz; PPTg: 25	12 months	UPDRS-III, Spatio-temporal gait measurements		PPTg and STN DBS were associated with changes in spatio-temporal and kinematics variables.
Schrader et al., 2013	1	66	20	Non-randomized, non-blind	Bilateral GPi and bilateral PPN	GPi: 130 Hz; PPN: 25 Hz	4 weeks	Computerized gait analysis		Combined stimulation markedly improved gait ignition and FOG.
Weiss et al., 2013	12	65	17.6 (10–26)	cross-over double-blind randomized controlled clinical trial	Bilateral STN and Bilateral SNr	STN: NA; SNr: 125 Hz	3 weeks	UPDRS-II, III, Freezing of Gait Assessment Course		Combined stimulation specifically improved FOG, whereas balance impairment remained unchanged.
Brosius et al., 2015	1	45	NA	double-blind, pseudo-randomized	Unilateral right STN and SNr interleaved DBS	15 or 125 Hz	No long term follow-up	Interrupted time series design		Unilateral right STN and SNr interleaved DBS significantly improved FOG.

*FCSRT, Free and Cued Selective Reminding tests; FOG, freezing of gait; FOG-Q, freezing of gait questionnaire; NFOG-Q, new FOG-Q; GPi, globus pallidus internas; MDRS, Mattis Dementia Rating Scale; MMSE, Mini Mental State Examination; MWSCT, Modified Wisconsin Card Sorting Test; RAVLT, Rey's Auditory Verbal Learning Test; ROCF, Rey–Osterrieth Complex Figure; RSGE, The Rating Scale for Gait Evaluation; S&E, Schwab and England scale; STN, subthalamic nucleus; PPN, pedunculopontine nucleus; DBS, Deep brain stimulation; PD, Parkinson's disease; SNr, substantia nigra pars reticulate; SWS, Stand-Walk-Sit; UPDRS, Unified Parkinson's Disease Rating Scale*.

### STN stimulation

STN is the common therapeutic target of DBS in PD. DBS can control the symptoms of tremor, tonic and bradykinesia in the long term and partially improve the gait, postural stability and articulation in the mid-term. However, the symptoms may deteriorate within 3–5 years after surgery (Moro et al., 2010a). PD patients suffer the most from FOG, which leads to loss of independence and mobility. Therefore, if STN-DBS is proved effective against FOG, the clinical application of STN-DBS will be promoted. Some recent small-sample-size trials are concerned with the efficacy of STN-DBS for FOG, and valuable findings have been described.

However, a few studies showed that STN-DBS failed to improve the symptoms of FOG (Stolze et al., 2001; Fasano et al., 2012; Rocchi et al., 2012), though the majority seems to arrive at an affirmative conclusion. It was indicated that the different stimulation frequencies of STN-DBS had a large impact on the effect. High and low frequencies of stimulation in STN-DBS may produce opposite effects in the treatment of FOG. However, no consensus has been reached concerning the efficacy of STN-DBS in FOG. A study enrolled 45 PD patients who had bilateral STN-DBS switched from chronic high-frequency stimulation (HFS) (130 Hz) to low-frequency stimulation (LFS) (≤80 Hz). However, 23 of 45 patients did not remain ON due to worsening of other symptoms. After a 4-year follow-up, no significant improvement was found in total motor UPDRS scores and axial and gait subscores (Sidiropoulos et al., 2013). Similarly, a randomized, double-blinded study revealed there was no statistical difference in the outcomes of the patients' gait complaints such as balance and freezing between HFS (130 Hz) and LFS (60 Hz) (Phibbs et al., 2014). Whereas, many literature reports indicated that LFS outperformed HFS in improving the symptoms of FOG. A case series demonstrated STN-DBS with 60 Hz early in the DBS programming course led to clinical efficacy in more advanced PD patients with levodopa responsive gait disturbance and FOG (Ramdhani et al., 2015). Xie et al.'s research showed switching the frequency of STN-DBS from 130 to 60 Hz immediately alleviated the FOG in both OFF and ON status and the effect lasted for at least 10 months (Xie et al., 2012). Meanwhile, it was found by a randomized, double-blinded study that compared with routine 130 Hz of STN, LFS with 60 Hz markedly improved FOG and axial and parkinsonian symptoms with the reduction of aspiration frequency and swallowing difficulty (Xie et al., 2015). In addition, other studies showed that HFS could improve FOG and other gait disorders. Some non-randomized prospective studies indicated bilateral STN-DBS with HFS was associated with significant improvement in FOG scores at 6 months and the sustaining effects at 12-month follow-up (Vercruysse et al., 2014). Importantly, in Niu et al.'s study, besides FOG, global cognitive assessment of neuropsychological function was also performed with the Mattis Dementia Rating Scale. They demonstrated bilateral STN-DBS improved FOG as well as neuropsychological function at 6 and 12 months after surgery, suggesting the improvement in neuropsychological function may be an important mechanism underlying the therapeutic effect of STN-DBS on FOG (Niu et al., 2012). A randomized and double-blind study demonstrated STN stimulation alone significantly improved gait disturbance evaluated by clinical performance tests, fear of falling ratings and posturography (Nilsson et al., 2009). Vallabhajosula and colleagues performed a quantitative study which compared different frequencies and voltages of STN-DBS. They found the postural control and gait characteristics were improved by HFS and LFS, which was similar and clinical changes were relatively small (Vallabhajosula et al., 2015). It was demonstrated that the effective frequency of STN for FOG still remained controversial. Small-sample studies support that LFS (usually 60 Hz) seems to be consistently effective in PD patients at the usual HFS to improve FOG as well as functions of speech, swallowing and other axial symptoms (Xie et al., 2017). A review of the existing studies does not negate the potential efficacy of HFS in FOG, and the disagreement between the studies may be due to the factors of sample size and follow-up duration.

Besides stimulation frequency, unilateral or bilateral stimulation also seems to play a role. Most of the studies arrive at an affirmative conclusion, and some researchers explore the differences between unilateral and bilateral simulation. Chenji et al. compared bilateral STN stimulation with unilateral stimulation using a randomized and double-blinded approach, and it was demonstrated that bilateral stimulation produced greater improvement in step length and double-limb support time than unilateral stimulation (Chenji et al., 2017). Another randomized and double-blind study also revealed bilateral STN-DBS with HFS greatly improved motor and gait complaints in PD patients. The particular finding is that right-sided stimulation might produce slightly greater improvement than left-sided stimulation (Lizarraga et al., 2016). Although these studies suggested the superiority of bilateral STN-DBS over unilateral STN-DBS, the efficacy for the two sides may be different in one patient as the degree of involvement differs between the two sides. Therefore, the use of different stimulation parameters (frequency and voltage) for each side may produce a better effect for FOG. This is only a conjecture and more studies are needed to confirm this. Furthermore, the duration of DBS for FOG is another important consideration. Acute stimulation seems to be effective to alleviate FOG related symptoms (Vallabhajosula et al., 2015; Lizarraga et al., 2016). Till now, most studies which arrive at an affirmative conclusion concerning the efficacy of STN-DBS are randomized and double-blind trials with a follow-up period shorter than 12 months. Studies concerning the effects of STN stimulation on FOG which have longer follow-up are required. Fasano et al. studied a series of 20 consecutive patients who received continuous stimulation for 8 years, and it was found that gait impairment evaluated by UPDRS-III (item 29) was improved by STN-DBS during the 8-year follow-up compared with the baseline with only a slight worsening of cognition (Fasano et al., 2010). A meta-analysis also indicates an improvement of gait and FOG by STN-DBS for more than 4 years in the Med-Off/Stim-On condition, but not in the Med-On/Stim-On condition (Schlenstedt et al., 2017). The long-term potential effect of STN-DBS on FOG needs to be further corroborated.

STN is the most intensively studied nucleus in relation to DBS in PD. However, most of the existing studies regarding the effect of STN on FOG in PD patients have a small sample size. The actual effect and working mechanism of STN still remain unknown. Evidences from the existing studies seem to indicate that bilateral STN LFS is better than HFS in improving the symptoms of FOG and the effect can last for at least 1 year. Though the overall safety of STN LFS is high, the risk of aggravating other symptoms of PD still exists. There are some case reports that affirm the positive effect of STN-DBS in improving the cognitive function of PD patients while relieving the symptoms of FOG. But more validation studies are needed in this respect. Further research efforts should be devoted to the following aspects: (1) Large sample size, multicenter, randomized, blind, controlled trials; (2) To determine which frequency is optimal without aggravating other symptoms of PD; (3) To determine different stimulation parameters for each of the two sides and to discuss whether stimulation at different time for different sides produces a better effect; (4) Long-term follow-up to assess the efficacy; (5) To assess the outcome of FOG using different methods, subjective and objective. (6) To further investigate whether it can improve cognition.

### PPN stimulation

As mentioned above, PPN, also known as PPTg, is related to gait and gesture regulation and plays a key role in axial deficits such as FOG and postural instability in PD patients. It is also significant for the cognitive circuit. PPN stimulation to 6-OHDA lesioned rats affects neuronal activity in both the STN and SNr, suggesting PPN-DBS has potential effects to alleviate gait and motor symptoms in PD (Park et al., 2014). Meanwhile, degeneration of PPTg can cause gait deficits, which is considered as a model of gait disorder. Stimulation to posterior PPTg can improve specific gait parameters in this model (Gut and Winn, 2015). These animal experiments indicate the potential efficacy of PPN stimulation for the treatment of gait disorder.

It was reported DBS to PPTg promoted a significant increase of glucose utilization in bilateral prefrontal areas associated with improvement of delayed recall, executive functions and working memory (Alessandro et al., 2010; Ceravolo et al., 2011). It is indicated that PPN stimulation can improve cognition of PD patients. However, there have been few clinical studies on PPN stimulation for FOG treatment, and the majority of the existing studies have a small sample size. The conclusions that are drawn concerning the efficacy and working mechanism of PPN stimulation are still controversial. Ferraye and colleagues performed a double-blind cross-over study which enrolled 6 PD patients with severe FOG and demonstrated PPN-DBS improved the duration of freezing episodes as well as falls related to freezing. However, the overall results had no significant changes during the double-blind evaluation (Ferraye et al., 2010). A meta-analysis provides evidence that PPN-DBS may improve FOG and falling after PD, which may depend on the duration of follow-up and types of outcome measures (Wang et al., 2017). Similarly, most of the reports on PPN stimulation for FOG treatment have obtained positive results. Unlike STN stimulation, it is still disputable as to which PPN stimulation approach is superior, unilateral or bilateral PPN stimulation. A randomized, double-blind study consisting of 6 PD patients revealed unilateral low frequency (50–70 Hz) PPN stimulation contributed to a marked reduction in freezing and falls in the ON and OFF medication states both at 3 and 12 months after PPN-DBS (Moro et al., 2010b). Mestre et al. demonstrated in another randomized, double-blind study that unilateral low frequency (60–70 Hz) PPN stimulation gave rise to improvement in FOG at 2 years of follow-up, while there were no significant changes at 4 years (Mestre et al., 2016). Based on these studies, unilateral PPN stimulation alleviated the symptoms of FOG. Bilateral PPN LFS was also reported to improve FOG symptoms. Acute PPN stimulation improved gait and balance but not akinesia scores. Chronic PPN stimulation significantly improved falls frequency (Thevathasan et al., 2010). Similarly, a randomized, double-blind, cross-over study also indicated combination of PPN-DBS and levodopa treatment produced a significant decrease of the freezing episodes and the frequency of falls (Welter et al., 2015). In addition, a study demonstrated improvement of FOG by unilateral or bilateral stimulation, which was associated with attenuation of alpha activity detected by electroencephalography, suggesting that PPN-DBS had the potential to improve cognition while improving FOG (Thevathasan et al., 2012). It seems that either unilateral or bilateral PPN stimulation is effective for treating FOG. As to the preferred frequency of stimulation, it is generally believed that low-frequency PPN has a better effect, but there is a lack of comparative study between high- and low-frequency PPN stimulation. Whether PPN-DBS can improve FOG symptoms in the long term is another question. The existing studies seem to indicate that the efficacy may be insignificant if the follow-up duration is prolonged (Mestre et al., 2016). However, this conclusion also requires further validation.

Taken together, PPN-DBS may be a good option for patients with severe axial motor deficits, especially for PD patients poorly responsive to STN (Fasano et al., 2015b). LFS is more favored for PPN. Unilateral or bilateral PPN-DBS seems to improve FOG in PD patients, and this effect may last for at least 2 years. But it remains unclear whether unilateral or bilateral PPN-DBS is safe and effective over the long term. For the current study, there may be more evidences indicating that PPN stimulation can improve cognition of PD patients, but whether it can concurrently improve FOG symptoms remains unclear. Large samples, multicenter, randomized, blind, controlled trials are needed to confirm this and to determine which approach of PPN-DBS is better, unilateral or bilateral. In addition, long-term follow-up is required to assess the efficacy. Whether the use of different stimulation parameters or approaches at different stages of the disease can achieve a better effect of FOG symptoms and improve cognition remains largely unknown.

### Combined stimulation

So far, the DBS stimulation of only a single nucleus, STN, PPN or GPi, cannot improve all symptoms of PD patients. Some researchers propose the combined stimulation of these nuclei to improve the majority of PD symptoms, including FOG and cognitive function. However, very few studies are concerned with the combined stimulation of FOG. A randomized, double-blind research revealed PPN-DBS (25 Hz) associated with standard STN-DBS (130–185 Hz) improved gait and postural items of UPDRS-III (Stefani et al., 2017). Similarly, Peppe et al. demonstrated in 5 PD patients that bilateral PPTg- (25 Hz) and STN-DBS (185 Hz) resulted in changes in spatio-temporal and kinematics variables evaluated by the optoelectronic system 12 months after neurosurgery (Peppe et al., 2010). Although these studies did not particularly mention the treatment effect on FOG, it was indicated that the combined stimulation of PPN and STN did improve gait disorder. In addition, a study of one PD patient showed isolated bilateral PPN or GPi stimulation had a mild impact on gait ignition and FOG, but combined stimulation had a marked effect, suggesting combined stimulation of PPN and GPi may be a promising option for FOG treatment (Schrader et al., 2013). Besides, Brosius et al. reported unilateral right STN and SNr interleaved DBS significantly improved FOG in a patient with advanced PD (Brosius et al., 2015). Meanwhile, a cross-over double-blind randomized controlled clinical trial which involved 12 PD patients showed that combined stimulation of bilateral STN and SNr specifically improved FOG, whereas balance impairment remained unchanged, as evaluated by UPDRS-II, III and FOG Assessment Course (Weiss et al., 2013). Apparently, the combined stimulation of PPN plus STN, PPN plus GPi, or STN plus SNr, may be useful for the treatment of FOG in PD patients, but its effect on cognition is still unclear. However, the conclusions need to be verified by large-sample high-quality clinical trials. The optimal combination of nuclei to be stimulated and the stimulation parameters should be determined.

### Side effects

Studies concerning the effects of DBS on FOG and cognitive impairment displayed safety of this treatment. The side effects of STN-DBS mainly resulted from the switching of stimulation frequencies. In one study that assessed the effects of DBS on FOG, 1 out of 20 patients dropped out due to a severe freezing episode when switching from HFS to LFS (Phibbs et al., 2014). Sidiropoulos et al.'s work revealed that approximately 3/4 of the total patients did not remain on LFS switched from HFS owing to the side effects including worsening of tremor, dystonia, gait and upper limb paresthesias (Sidiropoulos et al., 2013). Until present, limited studies are involved in evaluating the side effects of PPN-DBS. Welter and colleagues investigated the effects of PPN-DBS on FOG and cognitive function. They reported two patients with side effects of infection and midbrain hematoma (Welter et al., 2015).

## Conclusions and future perspectives

FOG is closely related to cognition in the pathogenesis. These are both common symptoms in the late stage of PD, which are difficult resolve and severely affect physical coordinate ability of the patients. DBS proves effective for FOG symptoms in PD patients, especially those with a long disease course and poorly responsive to medication. Considering the “cognitive model” as one of the underlying mechanisms of FOG, we speculate the effect of DBS to alleviate FOG may be associated with cognitive functions improvement. As shown in Figure 1, the cognitive model includes two tracks: a direct route requiring automatic responses regulated by the basal ganglia, and an indirect route eliciting a controlled response regulated by frontal cortical areas. DBS may modify the two routes via different targets. The automaticity and controlled processes in the indirect route may be regulated by STN-DBS. In addition, as close interconnections exist among the cerebral cortex, basal ganglia and PPN, it is possible that PPN-DBS is able to regulate both direct and indirect routes.

Based on the above hypothesis, this article reviewed relevant studies over the past 10 years and found that bilateral LFS of STN is a preferred option and that the treatment effect can last for at least 1 year. Although the overall safety of LFS stimulation of STN is high, there is still risk of aggravating other symptoms of PD. PPN stimulation is recommended for patients poorly responsive to STN stimulation. LFS is a preferred approach for PPN-DBS, and either unilateral or bilateral LFS can improve the symptoms of FOG with the efficacy lasting for at least 2 years. In theory, combined stimulation may achieve a better effect as it can improve various symptoms. The most intensively studied are the combined stimulation of PPN plus STN, PPN plus GPi, and STN plus SNr, all of which can achieve a satisfactory effect for FOG. Meanwhile, due to the close relation in mechanism, it is speculated that the mechanism of DBS treating FOG may be related to cognitive improvement. Given results of the current studies, PPN has greater potential to become the target of treating FOG and cognitive disorder. However, most of the existing studies have a small sample size and cannot reflect the real efficacy of DBS stimulation for FOG and cognition. It is also difficult to determine which nucleus can perform the optimal effect. Moreover, these studies only demonstrated the effects of DBS on FOG and cognitive impairment in PD patients through the clinical observations, lacking discussion of the mechanisms. Therefore, this review mainly summarizes the effects rather than the mechanisms. Large samples, multicenter, randomized, blind, controlled trials are needed to determine the optimal frequency and approach of stimulation and the optimal combination of nuclei to be stimulated through subjective and objective assessment of FOG and cognition as well as investigating the effect of DBS on the two. Long-term follow-up should be performed to assess the efficacy and to formulate the individualized DBS regimen for the patients. In addition, thorough investigation of the involved mechanisms may be another relevant research topic.

## Author contributions

CH, HC and YZ wrote and edited the manuscript. XW revised the review.

### Conflict of interest statement

The authors declare that the research was conducted in the absence of any commercial or financial relationships that could be construed as a potential conflict of interest.
